# Positioning of earphones and variations in auditory thresholds^☆^^☆☆^

**DOI:** 10.1016/j.bjorl.2015.08.016

**Published:** 2015-09-08

**Authors:** Bettina Poggi Almeida, Pedro de Lemos Menezes, Kelly Cristina Lira de Andrade, Cleide Fernandes Teixeira

**Affiliations:** a Especialização em Fonoaudiologia do Trabalho, Universidade Estadual de Ciências da Saúde de Alagoas (UNCISAL), Marechal Deodoro, AL, Brazil; b Física Aplicada à Medicina e Biologia, Universidade Estadual de Ciências da Saúde de Alagoas (UNCISAL), Marechal Deodoro, AL, Brazil; c Saúde da Comunicação Humana, Universidade Federal de Pernambuco (UFPE), Recife, PE, Brazil; d Saúde Coletiva, Universidade Federal de Pernambuco (UFPE), Recife, PE, Brazil

**Keywords:** Pure-tone audiometry, Auditory threshold, Occupational health, Audiometria de tons puros, Limiar auditivo, Saúde do trabalhador

## Abstract

**Introduction:**

One of the problems observed in pure-tone audiometry tonal has been the variation in test results of a same individual, particularly at frequencies of 4 kHz, 6 kHz and/or 8 kHz. Improper placement of headphones is one of the factors that can cause alterations in results.

**Objective:**

To compare differences in auditory thresholds using earphones positioned by the examiner and by the worker.

**Methods:**

Clinical and experimental study conducted in 2009, with 324 workers aged between 19 and 61 years, with a mean of 33.29 years and mean exposure time of 7.67 years. All subjects were familiar with audiometry procedures. Auditory thresholds were obtained at frequencies of 0.25–8 kHz, with earphones positioned by the examiners, and at frequencies of 4, 6 and 8 kHz, with earphones placed by workers in a comfortable position, following the examiner's instructions. The thresholds obtained in these two situations were compared.

**Results:**

The three frequencies exhibited better responses with earphones placed by the workers themselves (*p* < 0.001). At a frequency of 8 kHz a greater difference was found (*p* < 0.001), with a mean of 13.89 dB and standard deviation of 6.07 dB.

**Conclusion:**

Earphone placement by the workers themselves under supervision of the examiner results in improved mean auditory thresholds at frequencies of 4, 6 and 8 kHz, the last one significantly higher than the other two.

## Resumo

**Introdução:**

Um dos problemas observados na audiometria tonal é a variação nos resultados de testes de um mesmo indivíduo, sobretudo nas frequências de 4, 6 e/ou 8 kHz. A colocação indevida dos fones é um dos fatores que podem causar alterações nos resultados.

**Objetivo:**

Comparar as diferenças nos limiares auditivos com os fones posicionados pelo examinador e pelo paciente.

**Método:**

Estudo clínico e experimental realizado, em 2009, com 324 trabalhadores, com idade entre 19 e 61 anos, média de 29,33 anos e tempo médio de exposição ao ruído de 7,67 anos; todos familiarizados com os procedimentos da audiometria. Os limiares auditivos foram obtidos nas frequências de 0,25 a 8 kHz, com os fones colocados pelos examinadores; e nas frequências de 4, 6 e 8 kHz com os fones colocados pelos trabalhadores em posição de conforto, sob orientação do examinador. Os limiares obtidos nas duas situações foram comparados.

**Resultados:**

As três frequências apresentaram melhores respostas com os fones colocados pelos próprios indivíduos (p < 0,001). Na frequência de 8 kHz foi encontrada a maior diferença (p < 0,001), com média de 13,89 dB e desvio padrão de 6,07 dB.

**Conclusão:**

A colocação dos fones de ouvido pelos próprios trabalhadores, sob supervisão dos examinadores, resulta na melhora dos limiares auditivos médios nas frequências de 4, 6 e 8 kHz, sendo esta última significativamente maior que as demais.

## Introduction

Since pure-tone audiometry depends directly on the response of the individual, a number of variables must be controlled during the process. It must be conducted using standardized criteria, such as the cabin and equipment duly calibrated according to Regulatory Guideline No. 7 (NR7), annex I, item II, amended by Decree No. 19, of April 19, 1998, of the Department for Work Health and Safety of the Ministry of Labor and Employment.^1^ Thus, it is indispensable that there be an interval between tones to prevent the possible effects of a temporary change in threshold from falsifying the results of sequential audiograms, which provide data on progressive hearing loss over time.^2^

One of the problems has been the variation in pure-tone auditory thresholds observed, related to the change in test results of a same individual, particularly at frequencies of 4 kHz, 6 kHz, and/or 8 kHz. It is important to underscore that since 1965, a number of authors have shown that instability in hearing test results can be provoked not only by factors intrinsic to the individual, but also by those related to the methodology adopted.^3^, ^4^, ^5^, ^6^

The inadequate placement of earphones may cause erroneous alterations in results, owing to discomfort and consequent lack of attention paid by the workers themselves and/or effect of this poor positioning on acute frequencies when the supra-aural phone exerts pressure on the auricular pavilion.^7^ Additional factors may also affect the validity and reliability of the audiogram, such as lack of equipment calibration, inappropriate test setting, position of the worker during the test, inadequate instructions, false responses, and the learning effect.^8^

Studies that show the variability of audiometric test results between the exams of a same individual, mainly for frequencies between 4 kHz and 8 kHz, remain scarce. Some studies compare variations in audiometric thresholds between genders, age, time of employment, and occupational function.^9^ When the test is aimed at monitoring occupational hearing, it is important that this variability be minimized by obtaining accurate results, in order to avoid inaccurate hearing loss measurement, the respective consequence for the worker, and the costs of work-related injuries.

The sequential audiometric test, for occupational purposes, is used as an epidemiological surveillance instrument to detect and monitor job-related hearing alterations; its methodology must include quality control to minimize the interference of variables on the results of the same individual. The variations in auditory threshold observed between the test used as reference and the sequential test must be analyzed according to the worsening criterion established by Decree 19 of the Ministry of Labor. In this respect, comparison analysis defined by this Decree considers as suggestive of triggering and/or worsening of noise-induced hearing loss (NIHL) whenever comparison between the sequential and reference tests shows a difference between mean auditory thresholds, at frequencies of 3 kHz; 4 kHz, and/or 6 kHz greater than or equal to 10 dB HL, or worsening by more than 15 dB HL.^1^ According to the Decree, this is an indicator that adverse conditions in the environment are not controlled and that preventive actions must be taken.

In this context, different sequential test results hinder comparative analysis, and may indicate discrepant findings that preclude conclusive analysis or triggering or worsening of hearing loss not compatible with reality, and does not reflect the efficacy of the hearing protector. It is important to highlight that the audiometric test is used to preserve the health of exposed workers, and it is a decisive document in employee hiring or judicial disputes involving hearing damage.

Since 1974,^10^ the Department of Labor of the United States (Occupational Safety and Health Assessment) has stressed the importance of audiometric retests as a method of controlling possible errors in detecting auditory thresholds, suggesting immediate reassessment to ensure reliable results that do not compromise the efficacy of auditory monitoring, also favoring companies that use a safety and health management system based on international standards such as OHSAS 18001.

Thus, the aim of the present study was to compare pure-tone auditory thresholds, obtained by workers who positioned the earphones themselves, as per instructions from the audiologist, with those obtained when earphones were placed solely by the health-care professional. Additionally, the study aimed at observing variations in auditory thresholds obtained after a second assessment.

## Methods

This is a cross-sectional study conducted at two facilities specialized in occupational audiology and consultancy in the field of worker health in Recife, Brazil. The sample was composed of 324 workers (both sexes) from a wide range of job areas (call centers, printing, transport, food, steel, port, security, drivers, furniture, hydroelectric, among others).

To avoid selection bias, individuals whose audiograms showed conductive or mixed hearing alterations and/or presented with visible collapse of the external acoustic meatus (EAM) were excluded. Audiograms were considered normal when auditory threshold was less than or equal to 25 dB (dB HL).^1^ Furthermore, workers enrolled in a hearing loss prevention program, with more than one audiogram performed, and with a comparison of results showing response variations were selected.

To determine the auditory thresholds of the experimental group, the descending technique at 10 dB intervals was used until the individual no longer responded to the sound. At this intensity, the ascending technique at 5 dB intervals was used until the individual could once again detect the sound. In the first test, one of the participants was assessed at frequencies between 0.25 kHz and 8 kHz, with a minimum rest period of 14 h. The same criteria were adopted in the second test, but only frequencies between 4 kHz and 8 kHz were assessed. A duly calibrated GSI-64 audiometer, with TDH-50 earphones was used in the experiments, in addition to prior inspection of the external auditory canal and tympanic membrane, excluding cases of earwax blockage.

Earphones were placed alternately in tests one and two. For the first worker, in test one, the earphones were placed by the examiner, while in test two they were placed by the worker, under guidance of the examiner. The order was reversed for the next worker. Test and retest results were compared for intensity in dB HL and frequency, before and after earphone repositioning.

The order of frequency presentation, choice of ear, and order of retest were random for each individual, in order to eliminate interference from tiredness and learning.

Data analysis was conducted by absolute and percentage distribution of descriptive statistics measures, using the paired Student's *t*-test with unequal variances. The hypothesis of equal variances was carried out using Levene's *F*-test. Finally, ANOVA was applied to test differences between frequencies, and Tukey's test for pair wise comparison was used in order to observe possible significant differences between the frequencies studied. A significance level of *p* < 0.05 was established using SPSS v. 21. The study was approved by the Ethics Committee, under registration No. 199/09.

## Results

Participants were aged between 19 and 61 years, with a mean of 33.29 years and standard deviation of 10.41. Most (65.1%) had completed secondary school. Analysis of audiometric distribution, after confirmation of auditory thresholds, revealed that 75% exhibited auditory thresholds within the normal range and 25% had an altered threshold in at least one of the frequencies. Auditory threshold distribution by frequency, comparing the first and second exam, irrespective of who placed the earphones, showed no statistically significant differences, with a *p*-value of 0.456.

**Figure 1:**
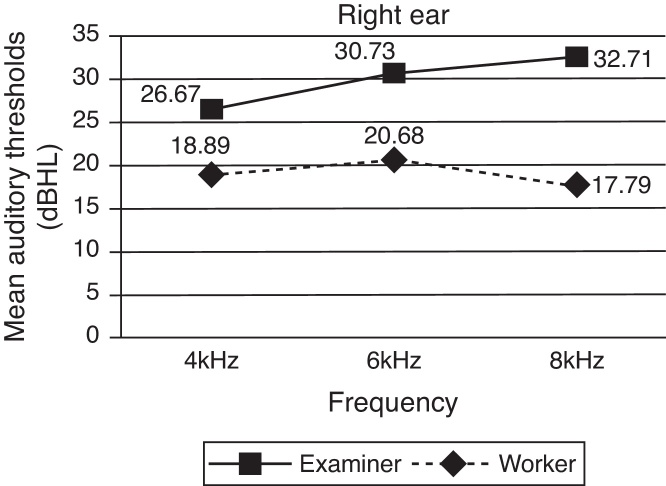
Distribution of mean auditory thresholds in the experimental group between tests and retests in the right ear.

**Figure 2:**
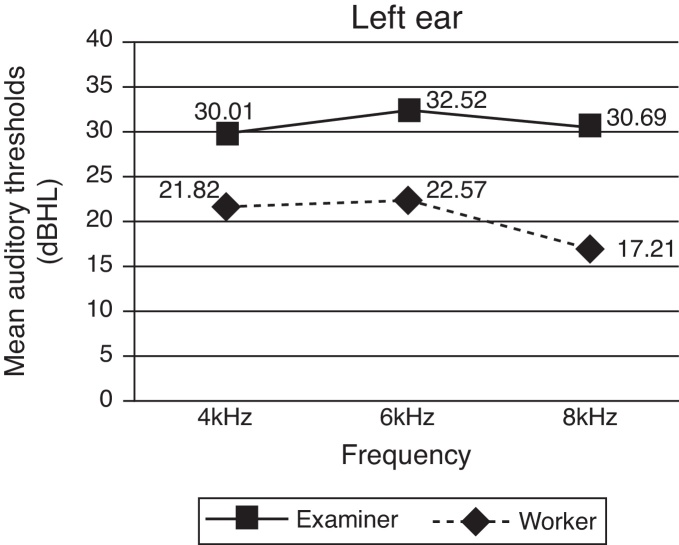
Distribution of mean auditory thresholds in the experimental group between tests and retests in the left ear.

The distribution of mean auditory thresholds in the experimental group between tests and retests, for both ears, was better after earphones were repositioned by the workers themselves, with a greater difference in intensity level for a frequency of 8 kHz, as shown in Figure 1, Figure 2.

Student's *t*-test for independent samples was applied to the results of both ears and showed no significant differences (*p* > 0.2). Thus, results were obtained for frequency, irrespective of which ear was tested.

Finally, the Student's *t*-test for paired samples, applied to the results obtained with the earphones placed by the examiner, and those with the earphones placed by the workers themselves according to examiner instructions, showed statistical differences for the three frequencies studied, with *p*-value < 0.001, as shown in Table 1.

**Table 1:** Mean standard deviation of altered and normal audiograms according to individual who placed the earphones.

Frequencies (kHz)	Hearing threshold
	Mean (dB) ± SD
*4* *kHz*	
Earphones placed by the examiner	28.42 ± 4.10
Earphones placed by the worker	20.52 ± 4.97
Difference	7.89 ± 2.53
*p*-Value	*p* < 0.001

*6* *kHz*	
Earphones placed by the examiner	31.60 ± 8.72
Earphones placed by the worker	21.52 ± 8.89
Difference	10.07 ± 5.30
*p*-Value	*p* < 0.001

*8* *kHz*	
Earphones placed by the examiner	31.39 ± 7.87
Earphones placed by the worker	17.50 ± 9.26
Difference	13.89 ± 6.07
*p*-Value	*p* < 0.001

The ANOVA test revealed the existence of statistically significant differences in frequencies depending on who placed the earphone (*p* < 0.001). Tukey's post hoc test, however, revealed significantly greater differences for 8 kHz than for the two others under study.

## Discussion

The first point to discuss is the choice of frequencies analyzed. It was decided to study only 4 kHz, 6 kHz, and 8 kHz, since they exhibit shorter wavelengths and are therefore more susceptible to interference because of earphone placement. Moreover, two of these, 4 kHz and 6 kHz, are very important in determining noise-induced hearing loss, according to Brazilian labor law (INSS/DAF/DSS No. 608, of August 5, 1998), whose text states that hearing loss predominates at frequencies of 6000, 4000, and/or 3000 Hz, progressing slowly at 8000, 2000, 1000, 500, and 250 Hz.^11^

The fact that workers are familiar with the annual audiometry test procedures and have high education levels may have minimized possible sample selection biases. Furthermore, the literature contains many studies that show no differences between auditory thresholds in tests and retests, when variables are controlled by familiarized workers, or when different earphones are placed by the examiner.^12^ Similarly, the results obtained in the present study showed no statistically significant differences between tests and retests, irrespective of who placed the earphones.

However, the present study sought to ensure earphone comfort when placed by the workers themselves, a key determinant for better sound transmission at short wavelengths, enhanced attention, and consequently improved auditory thresholds.^4^, ^5^, ^6^

In this respect, the findings of the present study corroborate those obtained in a clinical study showing significant differences in audiological tests and retests also at frequencies of 6 kHz and 8 kHz,^10^ and in other studies demonstrating collapse of the external acoustic meatus due to poor earphone placement.^13^, ^14^, ^15^, ^16^

The differences found between the two forms of earphone placement, in the two different tests, varied between 7.89 dB and 13.89 dB, corroborating studies with the same frequencies, which found differences between 15 dB and 20 dB for high-pitched sounds.^17^, ^18^

Finally, in relation to the other two frequencies studied, the differences in the 8 kHz frequency, which reached an average of 13.89 dB, may be related to its shorter wavelength, because the higher the frequency, the shorter its wavelength and the greater the interference, due to collapse or poor earphone placement.^8^

According to Ministry of Labor Decree 19, a variation of 10 dB or 15 dB must be considered when comparing between reference and sequential tests, since it indicates a significant change in auditory threshold, either triggering or exacerbating the disease; i.e., it allows the physician to infer that the individual is becoming ill. Thus, a false result compromises the prevention program established by companies to reduce the number of accidents, especially those put in practice in a health and safety management system in the workplace based on international standards, such as OHSAS 18001. These international standards use tools to systematically control and improve health and safety performance levels at work.^1^

Thus, contradictory results between sequential and reference tests must be avoided, since they may lead to incorrect interpretations of worker health, efficacy of the NIHL, and accidents, with all the consequences that these aspects represent to workers and the company.

Therefore, inadequate diagnosis of the auditory profile of workers, with an increase in false positive epidemiological results, may lead to an overestimation of the health risks faced by workers, thereby raising the accident rate in the company. According to the Brazilian Social Security Institute (Instituto Nacional do Seguro Social [INSS]), work-related hearing loss represents a technical epidemiological link, suggesting that the degree of workplace safety in a company is not satisfactory, which could increase social security contributions related to environmental risk.

Finally, given the limitations of the present study, owing to the specific group of workers, their familiarization with the exam, and high education levels, caution must be exercised when generalizing the results to other groups.

## Conclusion

Earphone placement by workers, under examiner supervision, resulted in better mean auditory thresholds at frequencies of 4 kHz, 6, kHz, and 8 kHz, the latter significantly higher than the other two.

## Conflicts of interest

The authors declare no conflicts of interest.
